# An innovative peptide toxicity prediction model based on multi-scale convolutional neural network and residual connection

**DOI:** 10.1093/bioinformatics/btaf462

**Published:** 2025-08-22

**Authors:** Shengli Zhang, Jingyi Ren, Yunyun Liang

**Affiliations:** School of Mathematics and Statistics, Xidian University, Xi’an 710071, P. R. China; School of Mathematics and Statistics, Xidian University, Xi’an 710071, P. R. China; School of Science, Xi’an Polytechnic University, Xi’an 710048, P. R. China

## Abstract

**Motivation:**

Peptide toxicity is a critical concern in the development of peptide-based therapeutics, as toxic peptides can lead to severe side effects, including organ damage, immune reactions, and cytotoxicity. Predicting peptide toxicity accurately is essential to ensure the safety and efficacy of these drugs.

**Results:**

In this study, we propose a novel model, ToxMSRC, to predict peptide toxicity using a combination of the continuous bag of words (CBOW) method from word2vec, synthetic minority over-sampling technique (SMOTE), multi-scale convolutional neural networks (CNN), and bidirectional long short-term memory (BiLSTM). This approach addresses the challenge of data imbalance by augmenting positive samples and improves feature extraction through multi-scale convolution. Furthermore, the model incorporates a residual connection that helps prevent overfitting and enhances generalization ability, improving classification performance. The model is evaluated on benchmark and independent test sets, achieving BACC scores of 92.17% on independent test1 and 86.89% on independent test2, outperforming existing state-of-the-art models. Additionally, ToxMSRC provides valuable insights into the relationship between peptide toxicity and amino acid sequences, demonstrating its potential and practical value in peptide-based drug development.

**Availability and implementation:**

The complete datasets, source code, and pre-trained models are made available at https://github.com/Renjingyi123/ToxMSRC and https://doi.org/10.5281/zenodo.15668530.

## 1 Introduction

Peptides, as promising therapeutic agents, have garnered significant attention due to their ability to target specific biological pathways with high precision (Rossino *et al.* 2023). They offer advantages over traditional small molecules, such as fewer side effects and more predictable pharmacokinetics (Wang *et al.* 2022). However, despite their potential, peptide-based drugs are not without risks, with toxicity being one of the most critical concerns in their development and application (Fetse *et al.* 2023). Peptide toxicity refers to the harmful effects peptides can exert on living organisms (Harris *et al.* 2015, Khan *et al.* 2018), which can manifest in various forms, including cytotoxicity, hemolysis, or immune responses (Cardozo *et al.* 2007). These toxic effects (Semwal *et al.* 2022) can result from several factors (Liebler and Guengerich 2005, Lee *et al.* 2020), such as the peptide’s structure, its interaction with cellular membranes (Lushchak *et al.* 2018, Chen *et al.* 2020), or its ability to trigger unwanted immune reactions (Saar-Dover *et al.* 2013). Toxic peptides may not only impair the function of healthy cells (Saar-Dover *et al.* 2013) but can also lead to serious side effects, including organ damage or even failure, which can be detrimental to the therapeutic potential of peptide-based drugs (Kanekura *et al.* 2018, Bhol *et al.* 2024). Given the complexity and variability of peptide toxicity, developing reliable and robust prediction models has become a key challenge.

Traditional experimental methods for peptide toxicity include in vitro cytotoxicity assays, acute toxicity testing (Garle *et al.* 1994), hematological and biochemical assays (Sirin *et al.* 2019), and immunotoxicity testing (Nagarkatti *et al.* 2010). These methods can effectively assess the toxicity of peptides on cells, tissues, and the entire organism (Gupta *et al.* 2013). However, they are often time-consuming, costly, and may involve animal experiments. Therefore, in recent years, machine learning-based prediction models have emerged as a complementary approach to these traditional methods, providing faster and more accurate toxicity predictions. In 2021, Wei *et al.* constructed ATSE (Wei *et al.* 2021), using graph neural networks and deep learning networks to process peptide sequence data in parallel, with the processed data being input as different heads into a multi-head attention mechanism for classification. Wei *et al.* constructed the ToxIBTL (Wei *et al.* 2022), which first pre-trained protein data using BLOSUM62. Convolutional neural network (CNN) and bidirectional gated recurrent unit (BiGRU) for data processing. Additionally, calculating graphical features, amino acid composition (AAC) and dipeptide composition (DPC) were used to process the data, which were then concatenated into new features. The features processed by both methods were combined for classification to build the model, which was further fine-tuned using peptide sequences in 2022. In 2013, Morozov *et al.* constructed the CSM-Toxin (Morozov *et al.* 2023) model, which uses a protein pre-training model to predict peptide sequences. In 2024, Rathore *et al.* proposed ToxinPred3.0 (Rathore *et al.* 2024), integrating motif-based, machine learning, and deep learning methods to improve the prediction of peptide toxicity, achieving promising results. Wang and Sung constructed a model named ToxTeller (Wang and Sung 2024), which includes four machine learning models for peptide toxicity prediction, namely logistic regression (LR), support vector machine (SVM), random forest (RF), and extreme gradient boosting (XGBoost). Jiao *et al.* integrated convolutional networks and Transformer models, and used a contrastive loss function to construct CAPTP (Jiao *et al.* 2024) for predicting peptide toxicity. The existing model, although already employing deep learning algorithms, still exhibits issues such as overfitting to some extent, resulting in relatively low recognition accuracy. Therefore, a combined approach incorporating residual connections, multi-scale CNN, and SMOTE is proposed to address these problems and improve accuracy.

In this study, we develop a new model framework, named ToxMSRC, which uses the continuous bag of words (CBOW) model from word2vec in the sequence feature encoding part. Unlike traditional handcrafted features and natural language processing methods, the word2vec-based approach can better learn the relationships between amino acids that are spaced apart. The encoded amino acid sequences are processed using the synthetic minority over-sampling technique (SMOTE) to handle positive samples in imbalanced data, doubling the dataset size to reduce overfitting to negative samples during subsequent learning. The processed sequences are then input into the multi-scale CNN to extract features at different scales, thereby reducing information loss. The extracted features are subsequently fed into a bidirectional long short-term memory (BiLSTM) for further feature extraction. Finally, the features extracted from both the CNN and BiLSTM are combined through a residual connection. This residual connection effectively prevents overfitting. Finally, the features are input into a fully connected layer for classification, resulting in improved classification performance. The existing model, although already employing deep learning algorithms, still exhibits issues such as overfitting to some extent, resulting in relatively low recognition accuracy. Therefore, a combined approach incorporating residual connections, multi-scale CNN, and SMOTE is proposed to address these problems and improve accuracy.

## 2 Materials and methods

### 2.1 Data description

Benchmark dataset plays a critical role in shaping the training outcomes and predictive performance of deep learning models. A high-quality dataset not only enables the model to learn more informative and robust features but also enhances its ability to accurately differentiate between positive and negative samples. To facilitate a better comparison of the performance of ToxMSRC, we use the same dataset as the CAPTP model. First, the datasets from CSM-Toxin, ToxinPred2 (Sharma *et al.* 2022), and ATSE are merged, and sequences with a length greater than 50 amino acids are removed. Next, in the UniProt (UniProt Consortium 2019) database, non-toxic peptide data is selected using the keywords “NOT KW-0800 AND NOT KW-0020 AND reviews: true.” and sequences containing non-standard amino acids or with a length exceeding 50 amino acids are excluded. The non-toxic peptides are then integrated with data from the three sources, and conflicting label samples are removed. Redundancy is eliminated using CD-HIT (Fu *et al.* 2012) software with a similarity threshold of 0.9 to remove redundant sequences, resulting in 2138 positive samples and 5375 negative samples. We use 85% of the data as the benchmark dataset and 15% as an independent test1. As shown in Table 1, the final training set consists of 1818 positive samples and 4569 negative samples, while independent test1 contains 320 positive samples and 806 negative samples. To further evaluate the model’s classification accuracy, an additional independent test2 was introduced, with 46 positive samples and 536 negative samples.

**Table 1.** Description of the benchmark dataset and independent dataset.

	Benchmark	Independent Test1	Independent Test2
Positive	1818	320	46
Negative	4569	806	536

### 2.2 Model architecture

**Figure 1.**
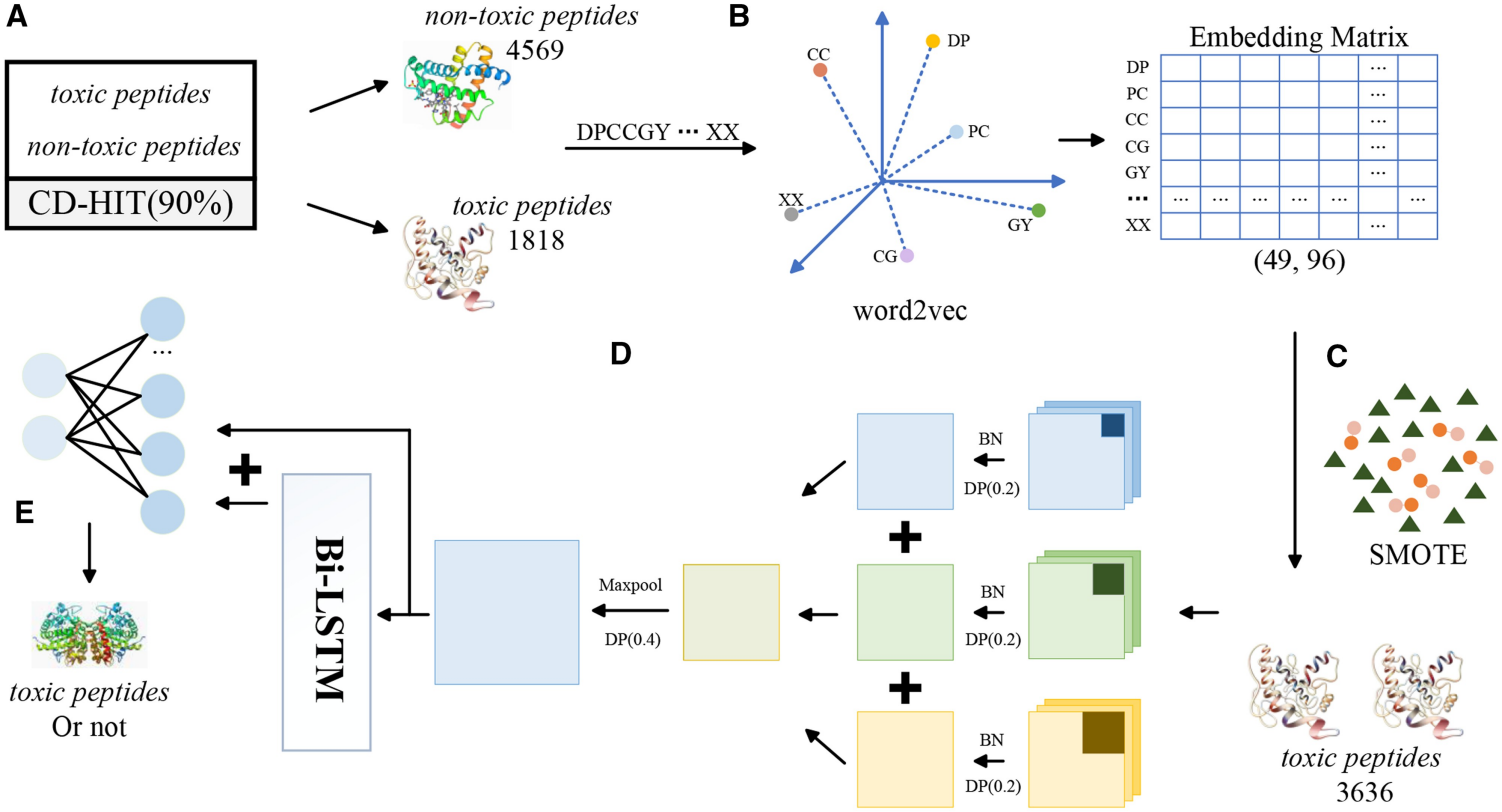
The overview of the ToxMSRC framework.

The ToxMSRC is comprised of six main modules: word2vec module, SMOTE module, multi-scale CNN module, BiLSTM module, classification module, and Fig. 1 shows the architecture of ToxMSRC. In the feature representation phase, we employ the CBOW method from word2vec to represent the amino acid sequences. The amino acid sequence is treated as a sentence, with adjacent pairs of amino acids considered as words for training, resulting in a feature matrix. Subsequently, the positive sample matrix is augmented using SMOTE to double the number of toxic peptide samples. In the feature extraction phase, the augmented positive and negative samples are input into a multi-scale CNN with kernel sizes of 3, 5, and 7 to extract information at different scales. The extracted features are then fed into a bidirectional long short-term memory (BiLSTM) for further feature extraction. The features output by both the multi-scale CNN and BiLSTM are combined through a residual connection and then input into a fully connected layer for classification, enabling the differentiation of toxic and non-toxic peptide sequences. During training, we set the learning rate (lr) to 0.0001 with 100 epochs and a batch size of 32. To prevent overfitting, we implement early stopping that terminates training when the validation metric fails to improve for 10 consecutive epochs. The training process of the parameters of the main layers is shown in Table 2.

**Table 2.** The parameters of ToxMSRC.^a^

Layers	Output shape	Parameters
input	[Batch, 49,96]	–
CNN-1	[Batch, 49,128]	Kernel size = 3, dropout ratio = 0.2
CNN-2	[Batch, 49,128]	Kernel size = 5, dropout ratio = 0.2
CNN-3	[Batch, 49,128]	Kernel size = 7, dropout ratio = 0.2
Max pooling1d	[Batch, 47,128]	Pool_size = 3, dropout ratio = 0.4
Conv1d	[Batch, 45,256]	Kernel size = 3
Bi-LSTM	[Batch, 45,256]	–
Flatten	[Batch, 11520]	–
Dense-1	[Batch, 128]	–
Dense-2	[Batch, 64]	–
Dropout	[Batch, 64]	Dropout ratio = 0.4
Dense-3	[Batch, 32]	–
Output	[Batch, 2]	Activation=“softmax”

a Each epoch takes an average of 28 s.

### 2.3 Word2vec module

Word2vec (Mikolov *et al.* 2013) introduced by Mikolov *et al.* in 2013, is a natural language processing technique that transforms words into dense vector representations within a continuous vector space, where semantically similar words are mapped to nearby points. The core concept of Word2Vec is to capture the semantic and syntactic relationships between words by utilizing the context in which they appear. Word2Vec consists of two model architectures: the CBOW model and the Skip-gram model. The CBOW model predicts a target word based on a context of surrounding words, while the Skip-gram model does the reverse, predicting the surrounding context words given a target word. Both models aim to optimize the same objective function, which is to maximize the likelihood of predicting context words from the target word. In this study, we select the CBOW model, as shown in Fig. 1B, treating each amino acid sequence as a sentence and considering each adjacent pair of amino acids as a word for model input. Since the peptide sequences in this study vary in length from 11 to 50 amino acids, all sequences are first padded with “X” to a length of 50 before being input to word2vec. For example, the sequence “DCCIIAGCPFGCTICC” is padded to “DCCIIAGCPFGCTICCX…X,” and then divided into [DC, CC, CI, …, XX] for feature representation. Each word is represented as a 96-dimensional vector, so each amino acid sequence is represented as a (49, 96) feature matrix.

Let the word vectors of the context words be represented by $v_{w}{\;}_{{\;}_{t-k}},K\ldots v_{w_{t}{\;}_{+k}}$, where $v_{w}$ is the vector representation of word w. The process is as follows:


(1) $$v_{c}=\frac{1}{2k}\sum_{j=t-k}^{t+k}v_{w_{j}}$$

(2) $$p(w_{t}|w_{t-k},\ldots w_{t+k})=\frac{\;\text{exp}(v_{c}^{T}v_{w_{t}})}{\sum {\;}_{w\in V}\;\text{exp}(v_{c}^{T}v_{w})}$$

where $v_{c}$ is the averaged context vector, $v_{w_{t}}$ is the word vector of the target word $w_{t}$. V is the vocabulary of all words.

### 2.4 SMOTE module

**Figure 2.**
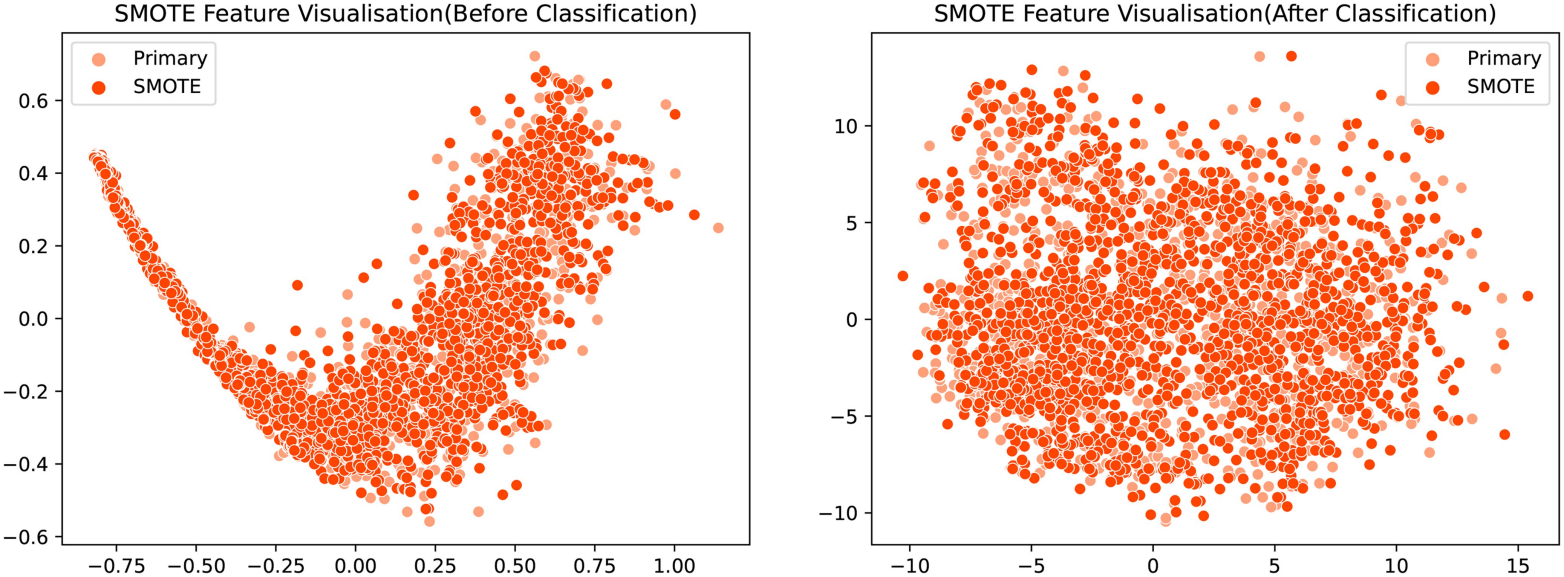
Visualization of positive Samples and SMOTE-sampled samples before and after training.

SMOTE (Chawla *et al.* 2002) introduced by Chawla *et al.* in 2002, is a technique designed to address the issue of class imbalance in datasets. In many classification tasks, particularly when one class has significantly fewer samples than the other, models tend to bias their predictions towards the majority class, leading to poor performance on the minority class. SMOTE works by generating synthetic samples for the minority class rather than simply duplicating existing samples. It achieves this by selecting a minority class sample and finding its nearest neighbors, then creating new synthetic samples through interpolation between the original sample and its neighbors in the feature space. This process increases the diversity of the minority class and helps improve model performance on imbalanced datasets. In this study, the number of toxic peptide samples is 1818, while the number of non-toxic peptide samples is 4569. Without balancing the dataset, the model would likely learn more from the non-toxic peptide samples, leading to reduced accuracy on positive samples. To prevent this issue, we apply SMOTE to handle the toxic peptide samples. For each sample, three nearest neighbors were calculated, and one was randomly selected to be added to the training set. As a result, during model training, the number of positive and negative samples is 3636 and 4569. The visualization in Fig. 2 confirms the robustness of our approach: while introducing some noise, SMOTE effectively preserves sample distribution patterns and boosts classifier accuracy.

### 2.5 Multi-scale CNN module

Fukushima proposed the concepts of convolutional and pooling layers in 1980. Subsequently, in 1998, LeCun *et al.* introduced the earliest deep convolutional neural network, LeNet-5 (LeCun *et al.* 1998), for handwritten digit recognition. In 2017, Krizhevsky *et al.* proposed AlexNet (Krizhevsky *et al.* 2017), which achieved groundbreaking results in large-scale image classification tasks through deep convolutional neural networks. CNN are composed of convolutional layers, pooling layers, and fully connected layers, with the convolutional layers being the primary component that extracts spatial features. The key advantages of CNN include parameter sharing, local connectivity, and the ability to learn multi-level feature representations, which enhance their efficiency and robustness in various machine learning tasks. In this study, the feature matrices trained by word2vec are input into convolutional layers with kernel sizes of 3, 5, and 7. Each convolutional layer is followed by a Batch Normalization (BN) layer and a dropout layer with a dropout rate of 0.2. The outputs of the convolutional layers are then summed to form a multi-scale convolutional layer. The features extracted by different kernel sizes vary in terms of receptive field size and the range of features captured. Smaller kernels focus more on local details, while larger kernels capture broader patterns and structural information. Therefore, utilizing multi-scale convolutions helps reduce information loss. Subsequently, the extracted features are passed through a max-pooling layer to highlight the most significant features and accelerate network training. The formula is as follows:


(3) $$\begin{array}{c} X_{\mathrm{out}}^{\mathrm{Conv}3}=\mathrm{ReLU}(\mathrm{Dropout}(BN(\mathrm{Conv}3(X))))\\ X_{\mathrm{out}}^{\mathrm{Conv}5}=\mathrm{ReLU}(\mathrm{Dropout}(BN(\mathrm{Conv}5(X))))\\ X_{\mathrm{out}}^{\mathrm{Conv}7}=\mathrm{ReLU}(\mathrm{Dropout}(BN(\mathrm{Conv}7(X))))\\ {X^{\prime }}_{\mathrm{out}}=X_{\mathrm{out}}^{\mathrm{Conv}3}+X_{\mathrm{out}}^{\mathrm{Conv}5}+X_{\mathrm{out}}^{\mathrm{Conv}7}\\ X_{\mathrm{out}}^{\mathrm{Conv}}=\mathrm{ReLU}(\mathrm{Conv}3(\mathrm{Dropout}(\mathrm{Maxpool}({X^{\prime }}_{\mathrm{out}})))) \end{array}$$

where X represents the output of the feature by word2vec.

### 2.6 BiLSTM module

BiLSTM (Schuster and Paliwal 1997), proposed by Schuster and Paliwal in 1997, is an extension of traditional LSTM networks designed to capture dependencies in sequential data by processing the input in both forward and backward directions. While a standard LSTM only considers past information (from left to right in the case of text or time series data), BiLSTM processes data in two passes one from left to right and the other from right to left, allowing it to capture both past and future context. This bidirectional approach enhances the model’s ability to understand complex patterns and relationships in sequential data, making BiLSTM particularly effective in tasks such as speech recognition, machine translation, and sentiment analysis. The formula is as follows:


(4) $$h_{t}^{f}={\text{LSTM}}_{f}(x_{t},h_{t-1}^{f})$$

(5) $$h_{t}^{b}={\text{LSTM}}_{b}(x_{t},h_{t+1}^{b})$$

where $x_{t}$ is the input at the time step t, $h_{t}^{f}$ and $h_{t}^{b}$ are the hidden states of the forward and backward LSTM at the times step t respectively. ${\text{LSTM}}_{f}$ and ${\text{LSTM}}_{b}$ are the forward and backward LSTM operations. Finally, the output of the BiLSTM at each time step is the concatenation of the forward and backward hidden states:


(6) $$X_{\text{out}}^{\text{BiLSTM}}=h_{t}=[h_{t}^{f},h_{t}^{b}]$$

### 2.7 Residual connection

Residual connection is a widely used technique in deep neural networks, introduced to address challenges such as vanishing or exploding gradients in deep networks. The core idea is to add skip connections between layers, allowing the output of a layer to be directly added to its input. This approach helps alleviate the issue of diminishing gradients during backpropagation, as it provides a direct path for the gradient to flow through. Residual connection enhances training by enabling information to flow more easily through deep networks, improving both training speed and model performance, particularly in architectures such as ResNet (He *et al.* 2016) and DenseNet (Huang *et al.* 2022). In this study, to prevent overfitting, we apply a residual connection between the output of the BiLSTM layer and the output of the convolutional layer. The formula is as follows:


(7) $$X_{\text{out}}=X_{\text{out}}^{\text{BiLSTM}}+X_{\text{out}}^{\text{Conv}}$$

### 2.8 Performance measures

In order to access the predictive capability of ToxMSRC, we use six evaluation metrics for evaluating the performance of the prediction model, including accuracy (ACC), sensitivity (SN), specificity (SP), balanced accuracy (BACC), area under the ROC curve (AUC) and Matthew’s correlation coefficient (MCC). The formulas are as follows:


(8) $$\begin{array}{c} \text{ACC}=\frac{\text{TP}+\text{TN}}{\text{TP}+\text{TN}+\text{FP}+\text{FN}}\times 100\%\\ \text{BACC}=\frac{1}{2}(\frac{\text{TP}}{\text{TP}+\text{FN}}+\frac{\text{TN}}{\text{TN}+\text{FP}})\times 100\%\\ \text{SN}=\frac{\text{TP}}{\text{TP}+\text{FN}}\times 100\%\\ \text{SP}=\frac{\text{TN}}{\text{TN}+\text{FP}}\times 100\%\\ \text{MCC}=\frac{\text{TP}\times \text{TN}-\text{FP}\times \text{FN}}{\sqrt{\left(\text{TP}+\text{FP})(\text{TP}+\text{FN})(\text{TN}+\text{FP})(\text{TN}+\text{FN}\right)}} \end{array}$$

where TN (True Negative) and TP (True Positive) represent the number of correctly identified non-toxic and toxic peptide sequences, FN (False Negative) and FP (False Positive) represent the number of non-toxic and toxic peptide sequences that are incorrectly identified. Since the training and testing datasets in this study are imbalanced, BACC and MCC are effective in evaluating the performance of imbalanced data. SN and SP refer to the accuracy of identifying all positive samples and the accuracy of identifying all negative samples, respectively. These evaluation metrics contribute to a better interpretability of the model in this study.

## 3 Results and discussion

### 3.1 Performance comparison of ToxMSRC with other models and model analysis

**Figure 3.**
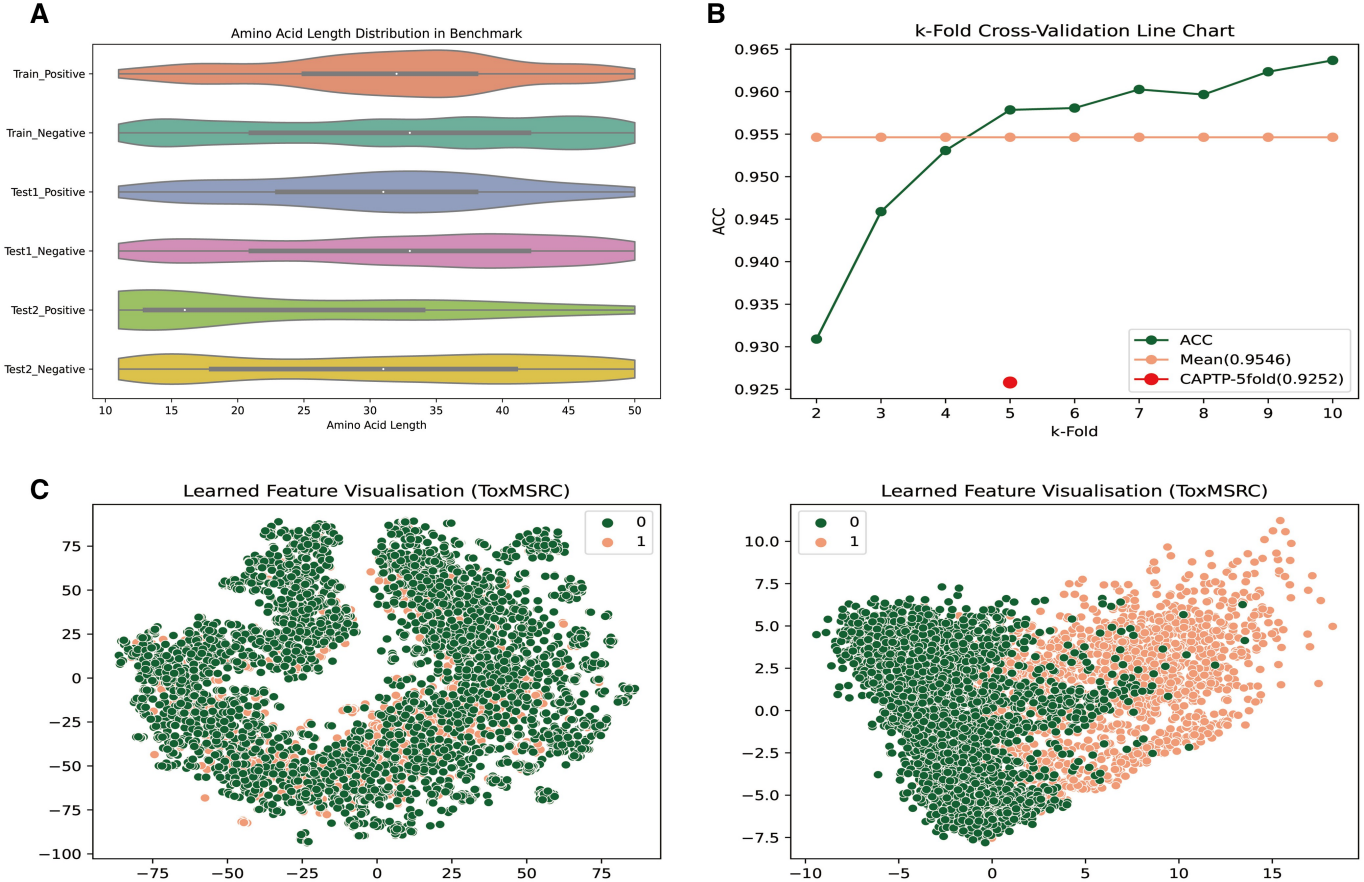
The overview of the ToxMSRC framework. Data analysis and performance of the ToxMSRC model on the benchmark dataset. (A) Peptide sequence length distribution of positive and negative samples in the benchmark dataset and two independent test sets. (B) Comparison and analysis of the 2–10-fold cross-validation results of the ToxMSRC model with the 5-fold cross-validation results from the CAPTP. (C) Visualization of PCA and t-SNE dimensionality reduction methods before and after training.

To evaluate the performance and generalization ability of the ToxMSRC model, we perform analysis and testing on both the benchmark dataset and the independent test sets. In the data analysis section, as shown in Fig. 3A, the length distribution of toxic and non-toxic peptide sequences across different datasets is illustrated. From Fig. 3A, we can observe that in the benchmark dataset, the lengths of toxic peptide sequences predominantly range from 25 to 40, while the lengths of non-toxic peptides are more uniformly distributed. In the independent test sets, we find that the sequence length distribution in independent test1 is similar to that in the training set, while the toxic peptide sequences in independent test2 tend to be shorter. As shown in the results in Table 3, we attribute the relatively better performance of the model on independent test1 to the high similarity in sequence length distribution between this test set and the training set.

**Table 3.** Performance comparison of ToxMSRC with other state-of-the-arts models.

Dataset	Method	ACC (%)	BACC (%)	AUC	Sn (%)	Sp (%)	MCC
Independent test1	CSM-Toxin	66.69	47.81	0.400	4.06	91.56	−0.076
	ToxinPred2	50.54	64.22	0.874	95.94	32.51	0.299
	ToxIBTL	91.56	91.56	0.916	91.56	91.56	0.803
	CAPTP	92.01	91.59	0.959	**90.63**	92.56	0.811
	**ToxMSRC**	**94.05**	**92.17**	**0.965**	87.81	**96.53**	**0.852**
Independent test2	ToxIBTL	87.80	78.47	0.785	67.39	89.55	0.431
	CAPTP	90.55	82.95	0.901	73.91	91.98	0.525
	**ToxMSRC**	**94.16**	**86.89**	**0.943**	**78.26**	**95.52**	**0.655**

The best results are highlighted in bold face.

In this study, to demonstrate the higher usability of the model, we compare several state-of-the-art models, as shown in Table 3. Given the significant imbalance between the positive and negative samples in the data, BACC and MCC provide a more reliable evaluation of the model’s performance. ToxMSRC achieves BACC and MCC scores of 92.17% and 0.852 on independent test1, outperforming the other models by at least 0.18% and 0.041, respectively. On independent test2, ToxMSRC achieves BACC and MCC scores of 86.89% and 0.655, surpassing the other models by at least 3.94% and 0.13%, respectively. Moreover, ToxMSRC also shows slightly higher performance than other models in terms of ACC, AUC, and SP, further proving the superior usability of our model. To provide an intuitive comparison of performance differences between ToxMSRC and other models, we conduct statistical analyses using Wilcoxon signed rank tests and Friedman tests to examine both pairwise differences between ToxMSRC and individual models as well as overall differences. On independent test1, the *P*-values between ToxMSRC and the other four models are 0.03125, 0.15625, 0.03125, and 0.03125, respectively, with an overall model *P*-value of 0.0118. On independent test2, the *P*-values compared with the other two models are both 0.03125, with an overall *P*-value of 0.0174. These results demonstrate the superior classification performance of our model.

Furthermore, as shown in Fig. 3B, the green line represents the training results of ToxMSRC on the benchmark dataset using 2–10-fold cross-validation, with the light pink line indicating the average cross-validation result of 0.9546. In contrast, the red dot represents the result of the currently best-performing model, after 5-fold cross-validation, which is 0.9252, significantly lower than the performance of our model. Figure 3C shows the visualization of the benchmark dataset after dimensionality reduction using PCA (Alain and Sylvie 2022) and t-SNE (Van der Maaten and Hinton 2008). It can be observed that the clustering effect of the data before and after classification is evident, demonstrating how the model’s feature extraction and classification process influences the separability of the data.

### 3.2 Exploration of the optimal architecture of ToxMSRC

To ensure that the methods used in our model are optimal, we conduct further analysis on the word2vec, multi-scale CNN, and residual connection in the model.

**Figure 4.**
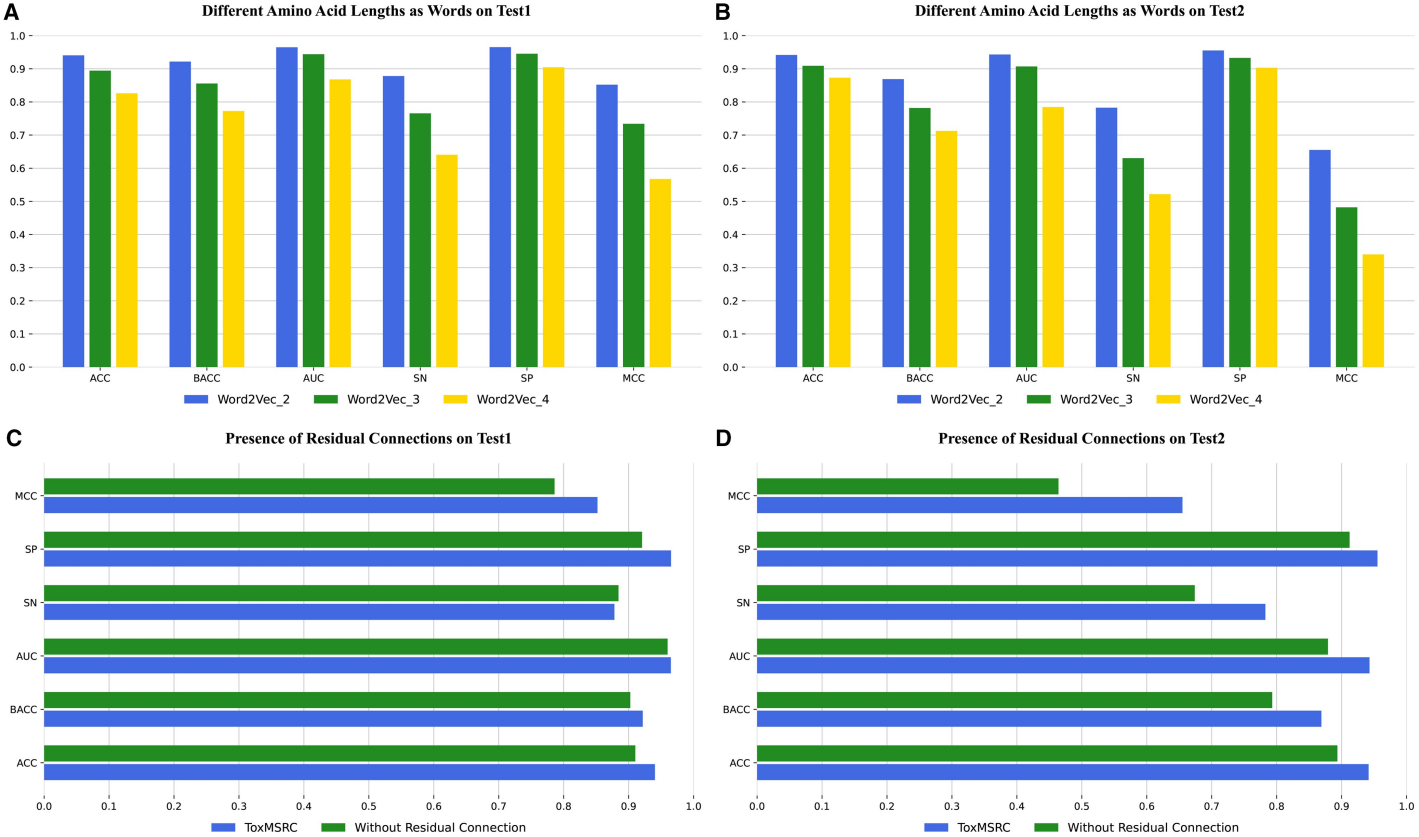
Comparison of different amino acid counts as words and the impact of residual connections.

In word2vec, we compare different lengths of amino acids as a word, as shown in Fig. 4A and B. We consider using three and four adjacent amino acids as one word for feature representation, resulting in feature matrices with dimensions of (48, 96) and (47, 96), respectively. From Fig. 4A and B, it is evident that as the number of amino acids represented by each word increases, all evaluation metrics gradually decrease. We believe that the short peptide sequence lengths and small dataset, especially the limited number of toxic peptides, contribute to this decline. A larger number of amino acids per word leads to less precise representation of each word’s features, resulting in poorer performance.

Therefore, using two amino acids as a word is the most reasonable choice for this study. Since the number of non-toxic peptides in our dataset is more than twice that of toxic peptides, we consider using SMOTE to augment the toxic peptide samples, ensuring that the model can adequately learn the information from the toxic peptide samples during the training process. To ensure the optimality of the augmentation quantity, Table 4 shows a comparison between doubling the data, not using SMOTE, and balancing the dataset. It is evident that when no balancing is performed, the accuracy of the negative samples is higher than that of the positive samples, indicating that the model has learned more features of the negative samples. When balancing is applied, although the accuracy of the positive samples increases significantly, the negative sample accuracy decreases notably due to SMOTE selecting nearby samples. We believe this is because when some samples select two nearby samples, the amount of similar data increases, which leads to a reduction in the accuracy of the negative samples. Therefore, doubling the positive samples is the optimal choice.

**Table 4.** Comparison of model performance with different data handling methods.

Dataset	Method	ACC (%)	AUC	Sn (%)	Sp (%)	MCC
Benchmark	Without SMOTE	99.08	0.996	97.96	99.52	0.977
	Balance SMOTE	97.93	0.995	98.29	97.79	0.950
	ToxMSRC	99.20	0.997	98.29	99.56	0.980
Independent test1	Without SMOTE	90.41	0.946	78.13	95.29	0.759
	Balance SMOTE	91.03	0.955	90.31	91.32	0.790
	ToxMSRC	94.05	0.965	87.81	96.53	0.852
Independent test2	Without SMOTE	91.58	0.893	69.57	93.47	0.533
	Balance SMOTE	88.83	0.890	73.91	90.11	0.484
	ToxMSRC	94.16	0.943	78.26	95.52	0.655

Generative adversarial networks (GAN) help address imbalanced sequence data by generating realistic samples for the minority class, improving data balance and model performance (Andresini *et al.* 2021). To better demonstrate the effectiveness of choosing SMOTE, we conduct a comparative analysis between samples generated by GAN and those generated by SMOTE. As shown in Table 5, the performance metrics on the training set and two independent test sets are consistently lower for GAN generated samples, especially in terms of BACC, where SMOTE outperforms GAN by over 2%.

**Table 5.** Comparison of different imbalance handling methods.

Dataset	Method	ACC (%)	BACC (%)	AUC	Sn (%)	Sp (%)	MCC
Benchmark	GAN	98.97	98.72	0.996	98.13	99.30	0.975
	SMOTE	99.20	98.93	0.997	98.29	99.56	0.980
Independent test1	GAN	91.20	88.78	0.954	83.13	94.42	0.782
	SMOTE	94.05	92.17	0.965	87.81	96.53	0.852
Independent test2	GAN	91.24	80.34	0.905	67.39	93.28	0.513
	SMOTE	94.16	86.89	0.943	78.26	95.52	0.655

**Figure 5.**
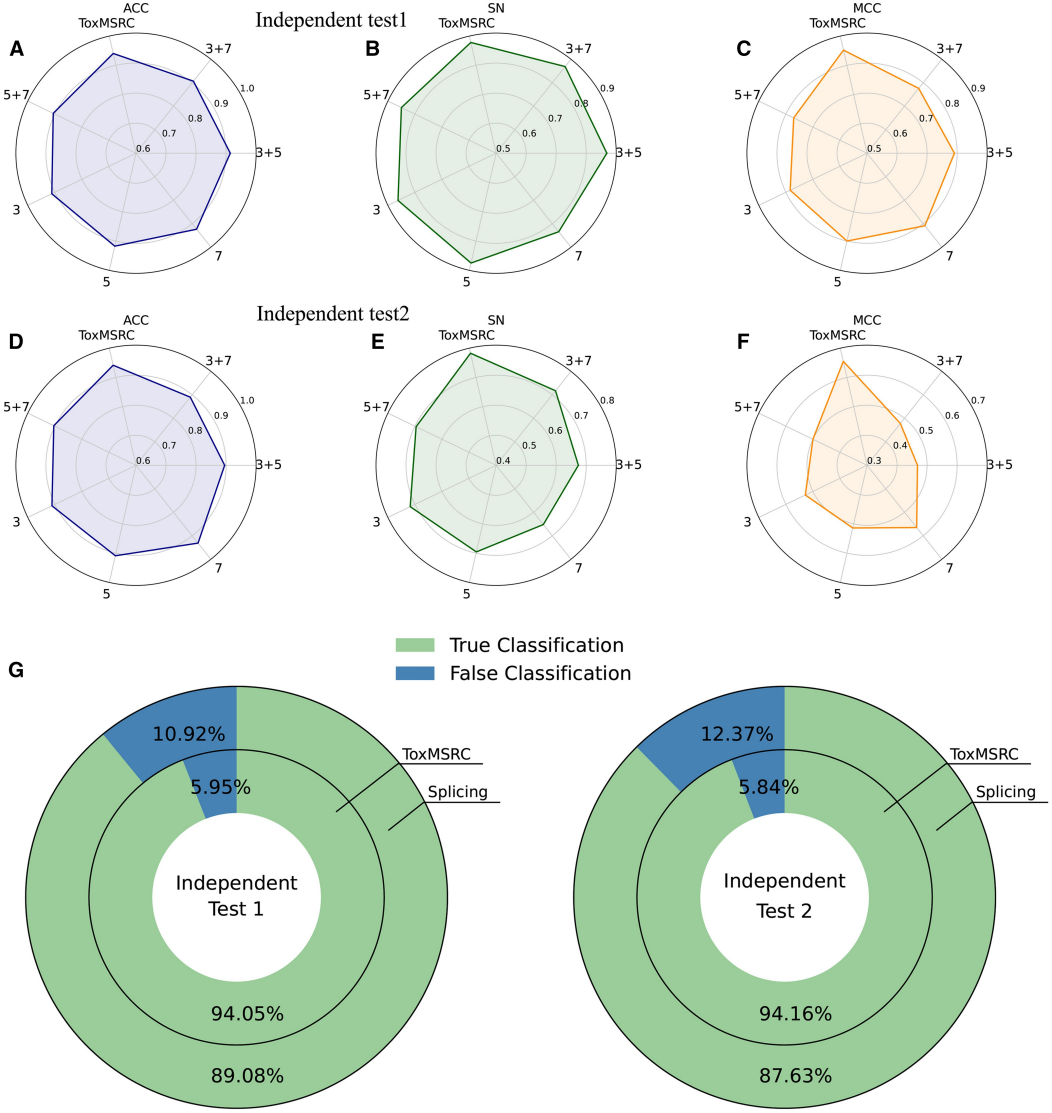
The impact of different convolutional kernel sizes on performance. (A–F) The ACC, SN, and MCC results of the multi-scale convolutional layer using only kernel sizes of 3, 5, 7, and combinations of 3 and 5, 3 and 7, 5 and 7 are presented on independent test1 and independent test2. (G) Comparison of ACC between matrix splicing and element-wise matrix addition in the final splicing part of the multi-scale convolution on independent test1 and independent test2.

In selecting convolutional kernel sizes for multi-scale convolution, we conduct a variety of analyses. As shown in Fig. 5A–F, we compare the ACC, SN, and MCC when using a single convolutional kernel size without multi-scale convolution. Additionally, we analyze the effects of combinations of kernel sizes 3 and 5, 3 and 7, and 5 and 7 on the two independent test sets. The results indicate that none of these combinations performs better than the combination of kernel sizes 3, 5, and 7. We believe that different convolutional kernel sizes extract features over different ranges: smaller kernels extract finer-grained features over smaller ranges, while larger kernels extract coarser features over broader ranges. When the peptide sequence length is shorter, smaller kernel sizes yield better results. For example, in independent test2, where toxic peptide samples are predominantly short sequences, the SN gradually decreases as the kernel size increases, as shown in Fig. 5E. In independent test1, where toxic peptides tend to have medium lengths, the SN is higher with a kernel size of 5, as shown in Fig. 5B. When combining different kernel sizes to form multi-scale convolution, our model selects kernel sizes that capture both fine details and broader features between amino acids, enabling the extraction of more comprehensive information. In addition, we also consider the impact of the method used to combine different scale convolutions on the model’s classification performance. Figure 5G shows that the outer ring represents the method of combining the multi-scale convolution output features using matrix splicing, while the inner ring represents the method of adding corresponding elements of the output feature matrices, similar to a residual connection. From the accuracy shown in the figure, the latter method achieves an accuracy improvement of 4.97% and 6.53% over the former.

In Fig. 4C and D, we analyze the importance of the residual connection in the model. Whether or not the residual connection is used, the model’s accuracy and cross-validation results during training are quite similar. However, on the two independent test sets, it is evident that the classification performance with the residual connection is higher than that without it. This indicates that by using the residual connection to input the data from the BiLSTM layer together with the previous data into the fully connected layer, the occurrence of overfitting in the model is significantly alleviated.

### 3.3 Performance comparison of ToxMSRC with other methods

**Figure 6.**
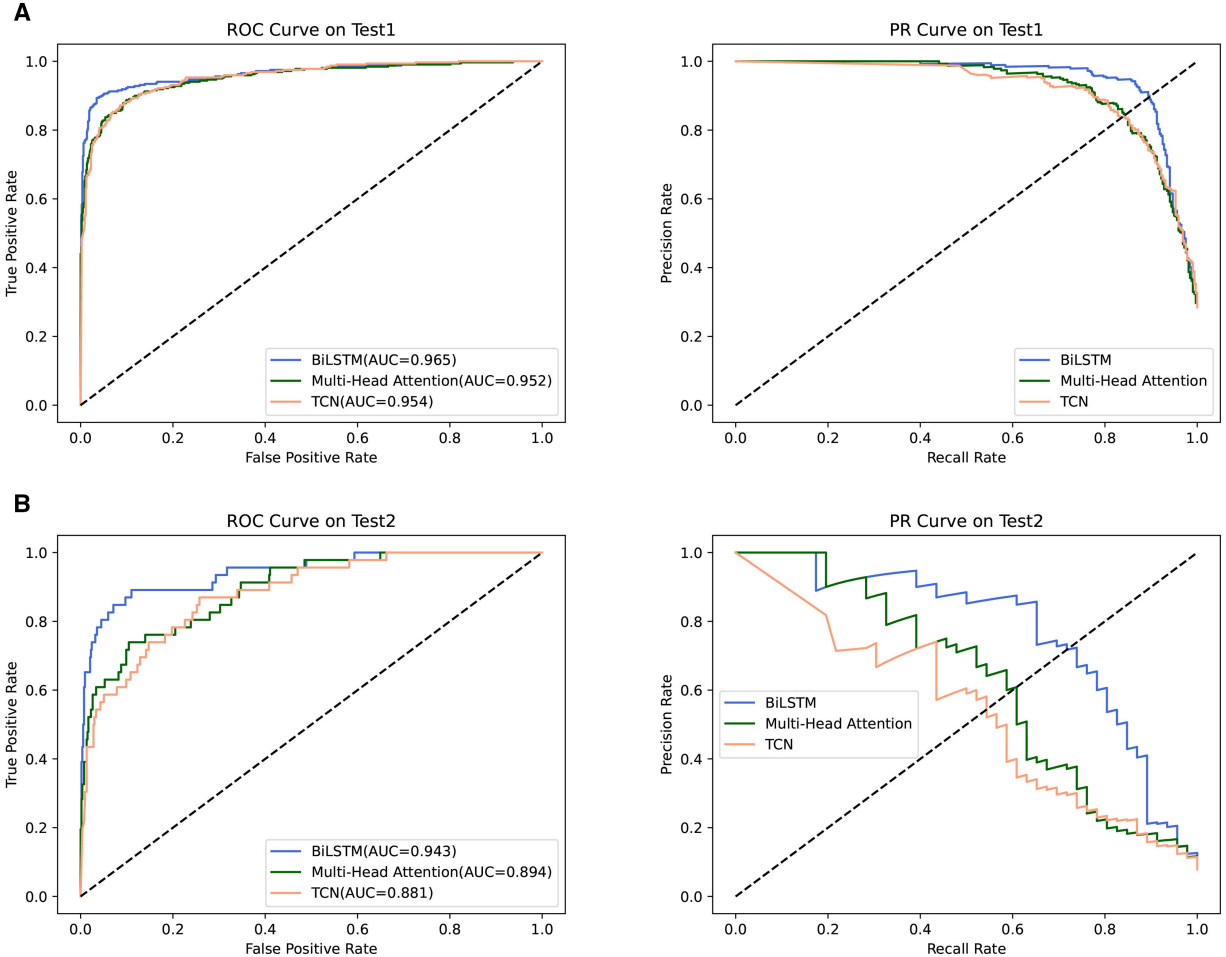
ROC and PR curves of different deep learning methods on independent test1 and independent test2.

Different methods extract different information and features. In this paper, we compare BiLSTM, multi-head attention (Vaswani *et al.* 2017), and temporal convolutional network (TCN) (Wan *et al.* 2022). As shown in Fig. 6, the area under the ROC curve (AUC) indicates model performance. The closer the ROC curve is to the top-left corner, the better the model’s performance. Similarly, the closer the PR curve is to the top-right corner, the better the model’s performance. The ROC and PR curves of these three methods indicate that the BiLSTM model selected at the end of this paper performs better.

### 3.4 Exploring the sequential characteristic of toxic peptides by using ToxMSRC

We use a computational approach similar to in silico mutagenesis (ISM) (Nair *et al.* 2022) to analyze the sequence features of peptide toxicity further. ISM is employed to determine the impact of each amino acid in the input sequence on the model’s prediction. This method has been widely applied in the sequence analysis of amino acids.

**Figure 7.**
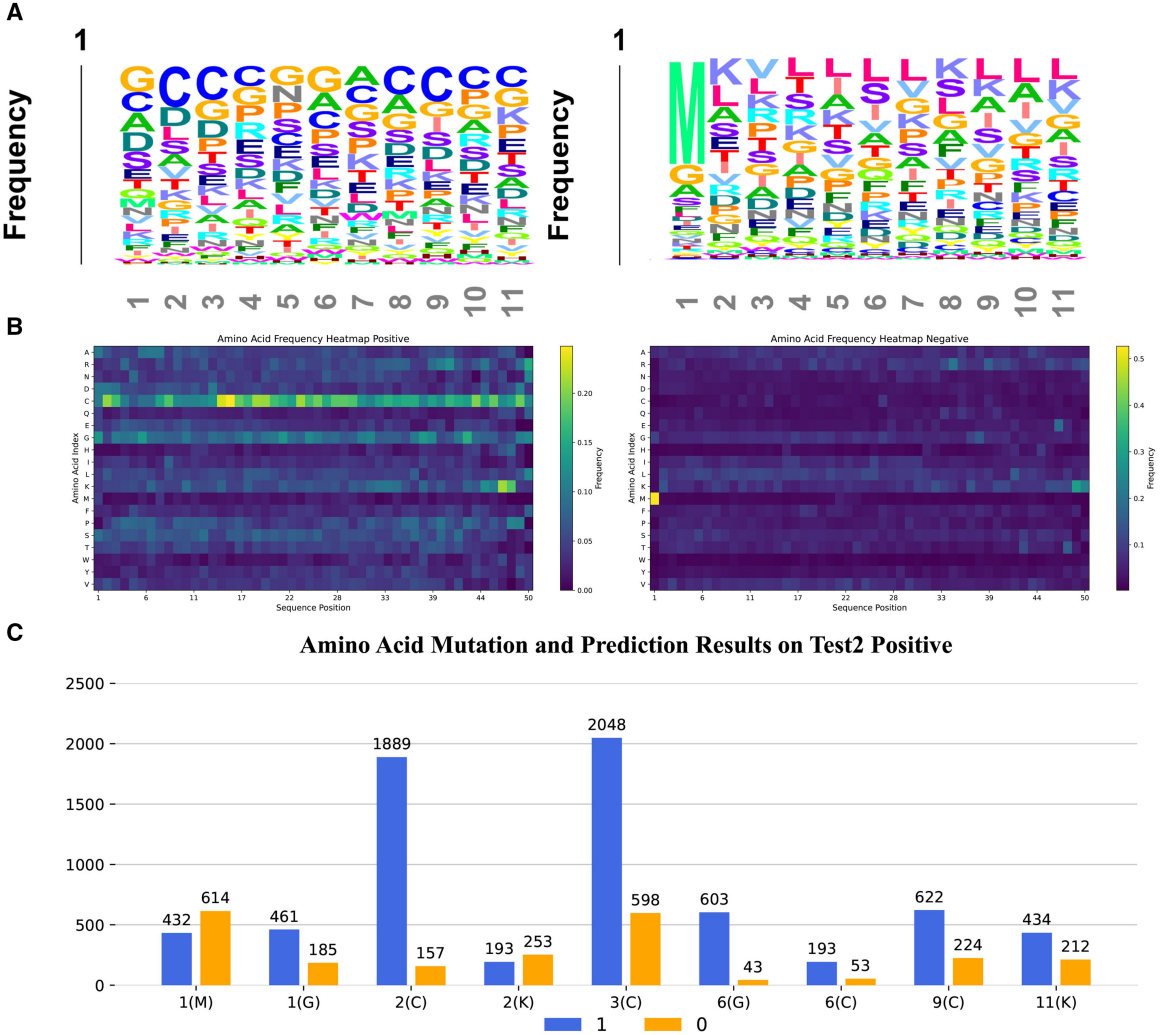
The result of exploring the sequential characteristics of toxic peptides using ToxMSRC. (A) The distribution of the proportion of amino acid counts at positions 1–11 for toxic peptides and non-toxic peptides. (B) The heatmap of the distribution of all amino acids for positive and negative samples in the benchmark dataset. (C) Prediction of toxic and non-toxic sequences for fixed amino acids at specific positions after 20 amino acid mutations (Positions 1–11) in toxic peptide sequences from independent test2.

Figure 7A shows the visualization of the amino acid frequency at positions 1–11 for toxic and non-toxic peptides in the benchmark dataset, while Fig. 7B displays the amino acid occurrence probabilities across all positions for both toxic and non-toxic peptides. It can be observed that, in positive samples, the amino acids Glycine (G) and Cysteine (C) appear more frequently. From the heatmap, it is evident that Cysteine (C) has the highest probability of occurrence at almost every position. In contrast, in negative samples, Methionine (M) predominantly appears at the first position, while the distribution of amino acids is almost uniform across other positions. During the model training process, these features are heavily utilized, ensuring the accuracy of the model’s classification.

In Fig. 7C, these findings are further validated. Figure 6C illustrates the mutation of 20 amino acids at positions 1–11 in 46 toxic peptide sequences from independent test2. This mutation results in a new dataset consisting of 10 120 amino acid sequence data points. By selecting a fixed position and a fixed amino acid, the sequences are input into the model to predict whether they are toxic or non-toxic, allowing us to track the counts of each classification. As shown in the figure, when the first amino acid in the sequence is Methionine (M), the model predicts a higher number of non-toxic sequences compared to toxic ones. When the first amino acid in the sequence is Glycine (G), the frequency of Glycine (G) in toxic sequences is more than twice that in non-toxic sequences in the training data. After mutation, the number of sequences with Glycine (G) at the first position predicted as toxic by the model exceeds the number of non-toxic sequences by more than twice, indicating that the model has effectively learned the sequence differences. Additionally, sequences with Cysteine (C) at positions 2, 3, 6, or 9 demonstrate a similar pattern. In the training data, Cysteine (C) rarely appears at these positions. Therefore, the mutated sequences have a much higher probability of being predicted as positive samples. Glycine (G) at position 6 occurs more frequently in toxic peptides than in non-toxic peptides in the training data. Consequently, after mutation, the predicted number of toxic sequences with Glycine (G) at position 6 follows the same trend observed in the training data.

Because these mutated samples are derived from toxic peptide sequences and each mutation only alters one amino acid, the model does not classify sequences based solely on one amino acid. Therefore, when training data contains amino acids that are more likely to appear in non-toxic sequences, the mutation of these amino acids may lead to less distinct classification differences. For instance, at positions 2 and 11, Lysine (K) is much more likely to appear in non-toxic peptides than in toxic peptides. At position 11, the occurrence probability is approximately the same for both toxic and non-toxic peptides. In the mutated data, the distribution of counts shows that at position 2, the number of non-toxic predictions for Lysine (K) exceeds the number of toxic predictions, although the difference is insignificant. At position 11, the number of positive samples exceeds that of negative samples. This further demonstrates the model’s accuracy in feature extraction.

### 3.5 Limitations and future directions

Although ToxMSRC demonstrates excellent performance in peptide toxicity prediction, it still has some limitations. Firstly, the model only applies to peptide sequences with lengths between 11–50 amino acids, and its generalization capability is limited to shorter or longer sequences. Additionally, the Word2Vec feature representation may not fully capture long-range dependencies. Future research could optimize the ToxMSRC model in several directions. On the one hand, more advanced data balancing techniques and feature extraction methods could be explored, such as using DeepInsight (Sharma *et al.* 2019) to convert sequence data into image data, in order to enhance the model’s robustness and prediction accuracy while extending its applicability to a wider range of peptide sequence lengths. On the other hand, developing an online prediction tool would facilitate the practical application of the model in peptide-based drug development, thereby advancing the field of precision medicine.

## 4 Conclusions

ToxMSRC provides an advanced and reliable solution to the critical challenge of predicting peptide toxicity, a primary concern for developing peptide-based therapeutics. Given the significant risks associated with toxic peptides, such as organ damage, immune reactions, and cytotoxicity, accurately predicting toxicity is vital for ensuring the safety of peptide drugs. The model integrates several innovative techniques, including the word2vec-based encoding method for capturing amino acid relationships, SMOTE for augmenting positive samples, and a multi-scale CNN for comprehensive feature extraction. Moreover, including a residual connection enhances model performance by reducing overfitting and improving generalization. The model’s outstanding performance on benchmark and independent test sets underscores its potential for guiding the design of safer peptide drugs. Additionally, the study incorporates a detailed analysis of amino acid sequence features and the role of mutations in toxicity prediction. Through computational approaches, such as ISM, the model’s ability to identify key sequence features contributing to toxicity was demonstrated. This further reinforces the potential of ToxMSRC in uncovering the underlying patterns in peptide toxicity. Ultimately, the model offers valuable insights for peptide drug development, highlighting the importance of accurate prediction models in mitigating toxicity risks and optimizing peptide-based therapeutic discoveries.

## Data Availability

The complete datasets, source code, and pre-trained models are made available at https://github.com/Renjingyi123/ToxMSRC and https://doi.org/10.5281/zenodo.15668530.
